# A conserved TLR5 binding and activation hot spot on flagellin

**DOI:** 10.1038/srep40878

**Published:** 2017-01-20

**Authors:** Wan Seok Song, Ye Ji Jeon, Byeol Namgung, Minsun Hong, Sung-il Yoon

**Affiliations:** 1 Division of Biomedical Convergence, College of Biomedical Science, Kangwon National University, Chuncheon 24341, Republic of Korea; 2 Division of Biological Science and Technology, Yonsei University, Wonju 26493, Republic of Korea; 3 Institute of Bioscience and Biotechnology, Kangwon National University, Chuncheon 24341, Republic of Korea

## Abstract

Flagellin is a bacterial protein that polymerizes into the flagellar filament and is essential for bacterial motility. When flagellated bacteria invade the host, flagellin is recognized by Toll-like receptor 5 (TLR5) as a pathogen invasion signal and eventually evokes the innate immune response. Here, we provide a conserved structural mechanism by which flagellins from Gram-negative γ-proteobacteria and Gram-positive Firmicutes bacteria bind and activate TLR5. The comparative structural analysis using our crystal structure of a complex between *Bacillus subtilis* flagellin (*bs*flagellin) and TLR5 at 2.1 Å resolution, combined with the alanine scanning analysis of the binding interface, reveals a common hot spot in flagellin for TLR5 activation. An arginine residue (*bs*flagellin R89) of the flagellin D1 domain and its adjacent residues (*bs*flagellin E114 and L93) constitute a hot spot that provides shape and chemical complementarity to a cavity generated by the loop of leucine-rich repeat 9 in TLR5. In addition to the flagellin D1 domain, the D0 domain also contributes to TLR5 activity through structurally dispersed regions, but not a single focal area. These results establish the groundwork for the future design of flagellin-based therapeutics.

Flagellin is a structural protein that assembles into the lash-like filament of a bacterial flagellum, which extends from the cell surface and allows bacteria to be motile^1^,^2^. Flagellin also promotes the adhesion and invasion of pathogenic bacteria into host cells as a virulence factor^3^. Because flagellin is exclusively found in bacteria and is one of the most abundant proteins in flagellated bacteria, flagellin is a main target of host immune surveillance. Upon bacterial invasion, flagellin is detected by Toll-like receptor 5 (TLR5) and NAIP5/NLRC4 in the host and activates innate immunity, contributing to the immediate clearance of pathogens from the host^4^,^5^,^6^,^7^.

TLR5 is an innate immune receptor located on the cell surface, and consists of an extracellular leucine-rich repeat (LRR) domain, a transmembrane domain, and an intracellular domain^7^. TLR5 recognizes flagellin as a pathogen-associated molecular pattern using the extracellular domain and activates the MyD88-dependent signaling pathway and NF-κB-mediated production of proinflammatory cytokines. Because flagellin functions as an activator for the first line of defense against flagellated pathogenic bacteria, flagellin has been characterized for the development of a vaccine carrier protein or a vaccine adjuvant^8^. Fusion proteins of flagellin with an antigen have been proven to be effective as experimental vaccines against diverse infectious diseases, including influenza, West Nile fever, malaria, plague, and tuberculosis^9^,^10^,^11^,^12^,^13^. Flagellin-activated TLR5 also protects hematopoietic cells and the gastrointestinal tissue from radiation and was reported to affect cancer cell survival and growth^14^,^15^,^16^.

Flagellin contains two to four domains. For example, *Bacillus subtilis* Hag flagellin, *Pseudomonas aeruginosa* type A FliC flagellin, and *Salmonella enterica* subspecies *enterica* serovar Typhimurium FliC flagellin have two (D0 and D1), three (D0, D1, and D2), and four (D0, D1, D2, and D3) domains, respectively. The common D0 and D1 domains are buried in the core of the flagellar filament by mediating inter-flagellin interactions and are conserved among bacterial species due to their functional importance in filament formation^17^,^18^,^19^. The D0 and D1 domains are believed to be the primary TLR5 stimulator because flagellin monomers, but not polymerized flagellins in the filament, activate TLR5^20^. In three- and four-domain flagellins, the D1 domain is extended to ancillary domains (D2 and D3) located on the surface of the flagellar filament and makes little or no contribution to filament formation^21^. In contrast to D0 and D1, the D2 or D3 domains exhibit substantial variation in sequence and structure and are considered to activate adaptive immunity and cause the undesirable toxicity of flagellin-based therapeutics. Thus, CBLB502, a D0/D1-containing anti-radiation bio-drug, was developed by removing the hypervariable domains (D2 and D3) from *Salmonella* flagellin^14^. Many Gram-positive bacteria, such as *Bacillus subtilis* and *Clostridium difficile*, express flagellin that lacks the hypervariable domains and thus contains the minimal regions (D0 and D1 domains) required for TLR5 activation and for flagellin polymerization into the flagellar filament^22^.

TLR5 responds to flagellins from Gram-negative β-/γ-proteobacteria and Gram-positive Firmicutes bacteria^20^,^23^. The flagellin-TLR5 interaction and its cellular outcome have been extensively studied using *Salmonella* flagellins. A structural and biochemical study of a complex between *Salmonella enterica* subspecies *enterica* serovar Dublin flagellin D1-D2 domains (*sd*flagellin^D1-D2^) and the N-terminal fragment of zebrafish TLR5 revealed that flagellin and TLR5 form a 1:1 complex via ‘primary binding’ and further homodimerize into a 2:2 complex via ‘secondary dimerization’^21^,^24^. However, due to the variations in the sequences and domains of flagellins, it is questionable whether numerous flagellins use the TLR5-recognition mechanism observed for *Salmonella* flagellin. Moreover, despite our knowledge of the flagellin-TLR5 binding interface, the contribution of each flagellin residue to TLR5 binding and activation has not been elucidated. The lack of this information impedes our efforts to develop new flagellin-based therapeutics through protein engineering.

To reveal the common mechanism by which flagellins from diverse bacterial species recognize and activate TLR5, we performed structural and mutational studies of the flagellin-mediated TLR5 interaction and activation. Our extensive comparative analysis demonstrates that an arginine residue in the flagellin D1 domain is a conserved TLR5 binding and activation hot spot by providing structural and chemical complementarity to the LRR9 loop of TLR5.

## Results

### TLR5 activation and binding by flagellins

**Figure 1:**
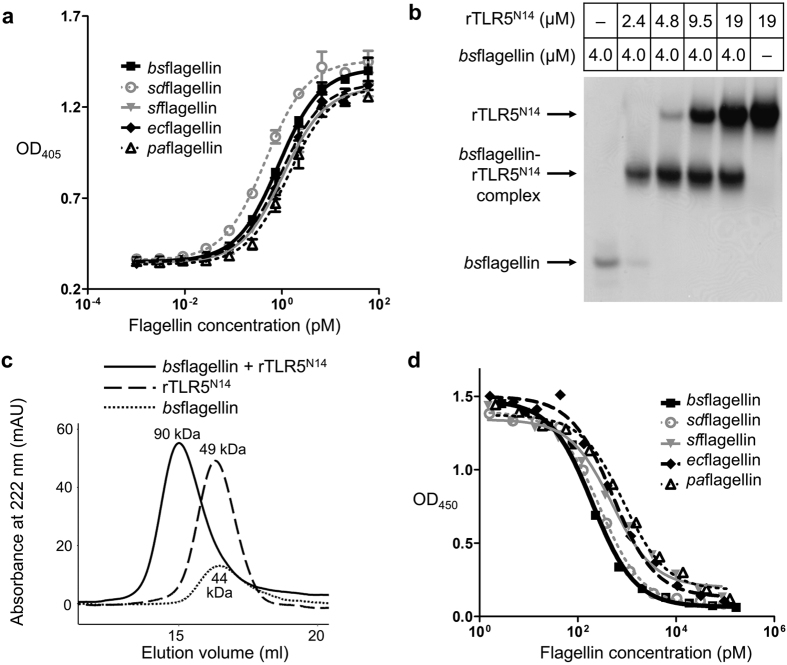
TLR5 interaction and activation by flagellins. (**a**) TLR5 signaling activities of *bs*flagellin, *sd*flagellin, *pa*flagellin, *sf*flagellin, and *ec*flagellin. Activities were determined in duplicate using the HEK293^TLR5^ reporter cell assay. The data [means ± standard deviation (S.D.); n = 2] are representative of three independent experiments that yielded similar results (Table 1). (**b**) Native PAGE analysis of the direct interaction between *bs*flagellin and rTLR5^N14^. (**c**) Gel-filtration analysis of the complex formation between *bs*flagellin and rTLR5^N14^. The apparent molecular weight of each peak was estimated using the elution volumes of gel-filtration standards and is shown near the peak. Protein elution was monitored by optical absorbance at 222 nm instead of 280 nm because *bs*flagellin does not contain any tryptophan and tyrosine residues that are detectable with 280 nm absorbance. (**d**) rTLR5^N14^-binding capacity of *bs*flagellin, *sd*flagellin, *pa*flagellin, *sf*flagellin, and *ec*flagellin determined with the competitive binding assay. The data shown are representative of at least three independent experiments that yielded similar results (Table 1).

To determine and compare the TLR5-activating abilities of diverse flagellins, an array of flagellins from Gram-negative γ-proteobacteria [*S. enterica* subspecies *enterica* serovar Dublin (*sd*flagellin), *Escherichia coli (ec*flagellin), *Shigella flexneri (sf*flagellin), and *P. aeruginosa (pa*flagellin)] and Gram-positive *B. subtilis (bs*flagellin) was prepared as recombinant proteins, and their TLR5 signaling activities were assessed with a reporter cell assay using human TLR5-transfected HEK293 (HEK293^TLR5^) cells containing an NF-κB-inducible secreted embryonic alkaline phosphatase (SEAP) reporter gene. The five flagellins strongly and comparably activated TLR5 signaling in a dose-dependent manner, with EC_50_ values of 0.57–1.68 pM (Fig. 1a and Table 1).

To confirm that the flagellin-mediated TLR5 activation occurs through a direct interaction between flagellin and TLR5, the formation of the flagellin-TLR5 complex was monitored by native PAGE and gel-filtration chromatography analyses using a recombinant TLR5 protein (rTLR5^N14^) that contains the LRR N-terminal capping motif (LRRNT) and LRR modules 1–14 of zebrafish TLR5. The rTLR5^N14^ protein is the only available recombinant TLR5 protein and was previously utilized for structural and biophysical studies of the *sd*flagellin-TLR5 interaction^21^,^25^. As for *sd*flagellin, all tested flagellins were able to bind rTLR5^N14^. In the native PAGE analysis, flagellin altered rTLR5^N14^ mobility and the mobility shift was complete at a molar ratio of 1:1 (Fig. 1b). In gel-filtration chromatography, flagellin formed a 1:1 complex with rTLR5^N14^ (Fig. 1c).

To examine whether different flagellin proteins induce TLR5 activation through the common binding site, a competitive binding assay was performed (Fig. 1d). *bs*flagellin was immobilized on the surface and incubated with rTLR5^N14^ for complex formation, which was competitively inhibited by simultaneously adding soluble flagellin. The five flagellins exhibited IC_50_ values of 204–673 pM, suggesting that they employ a common molecular pattern for TLR5 binding and possess comparable TLR5-binding affinity. Taken together, these results lead us to conclude that TLR5 similarly binds and responds to diverse flagellins from γ-proteobacteria and Firmicutes, potentially through a conserved mechanism.

### Crystal structure of the bsflagellin-TLR5 complex

**Figure 2:**
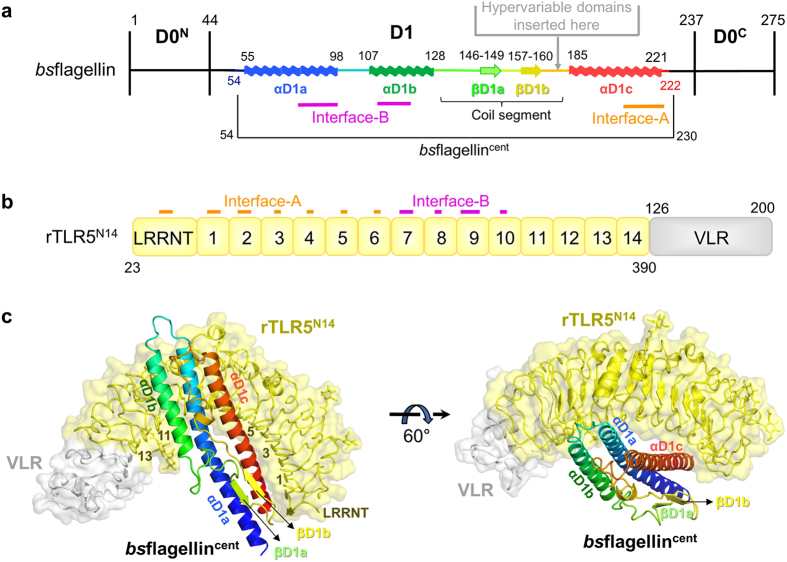
Crystal structure of the *bs*flagellin^cent^-rTLR5^N14^ complex. (**a**) Domain organization of *bs*flagellin. *bs*flagellin consists of two domains, D0 and D1. The D0 domain is divided into D0^N^ and D0^C^ by the insertion of the D1 domain. In three- or four-domain flagellins, hypervariable domains are located between the coil segment and the αD1c helix of the D1 domain. *bs*flagellin residues 54–222 were built in the *bs*flagellin^cent^-rTLR5^N14^ complex structure and are rainbow-colored from blue to red with secondary structure assignments. *bs*flagellin residues in binding interface-A and interface-B are schematically indicated by orange and magenta lines, respectively. (**b**) Schematic representation of the rTLR5^N14^ LRR modules (zebrafish TLR5 LRRNT-LRR14, yellow) fused to the C-terminal fragment of the hagfish variable lymphocyte receptor (VLR, gray). Binding interface-A and interface-B are delineated by orange and magenta lines, respectively. The LRR modules of rTLR5^N14^ are labeled accordingly. (**c**) Overall structure of the complex between *bs*flagellin^cent^ and rTLR5^N14^. rTLR5^N14^ is depicted in yellow ribbons with a transparent yellow surface representation, and its fusion partner, VLR, is colored in gray. *bs*flagellin^cent^ is shown in the rainbow-colored ribbon diagram from the N-terminus in blue to the C-terminus in red.

The previously reported crystal structure of a complex of rTLR5^N14^ with *sd*flagellin^D1-D2^ visualized the TLR5 interaction with a Gram-negative γ-proteobacterial flagellin containing both hypervariable and conserved domains^21^. In contrast to *sd*flagellin, many flagellins from Gram-positive bacteria, including *bs*flagellin, possess only the constant D0 and D1 domains and lack the hypervariable domains. *bs*flagellin binds and stimulates TLR5 in a manner similar to other flagellins that additionally contain hypervariable D2 and/or D3 domains, such as *sd*flagellin, *ec*flagellin, *sf*flagellin, and *pa*flagellin (Fig. 1). To reveal the structural mechanism of TLR5 binding to Gram-positive bacterial flagellin that lacks the hypervariable domains, the crystal structure of rTLR5^N14^ in complex with the central region of *bs*flagellin (*bs*flagellin^cent^; D1 residues 54–230) was determined at 2.1 Å resolution (Fig. 2 and Supplementary Table S1). Consistent with the biophysical assays performed in solution (Fig. 1b,c), *bs*flagellin^cent^ formed a 1:1 complex with rTLR5^N14^ in the crystal structure (Fig. 2c).

**Figure 3:**
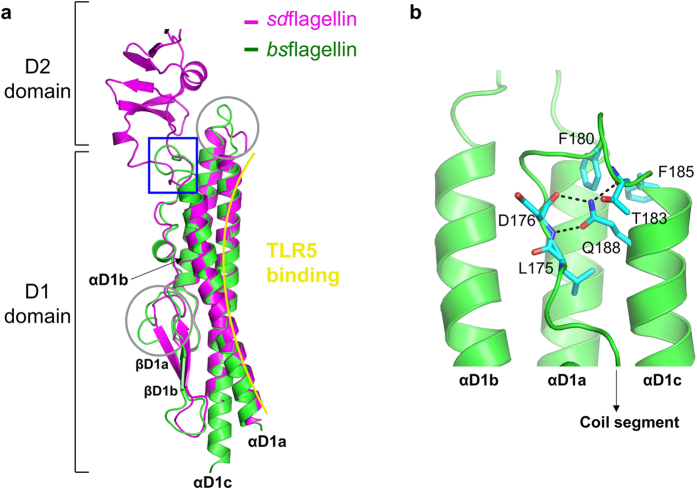
Flagellin structure in the *bs*flagellin^cent^-rTLR5^N14^ complex. (**a**) Structural comparison of *bs*flagellin^cent^ (green ribbons) and *sd*flagellin^D1-D2^ (magenta ribbons, PDB ID 3V47). The unique structural features of *bs*flagellin^cent^ are highlighted by a blue rectangle and gray circles. A sharp turn between the coil segment and αD1c helix in the *bs*flagellin^cent^ structure replaces the hypervariable D2 domain of *sd*flagellin^D1-D2^. (**b**) Close-up view of the blue rectangle in Fig. 3a.

The structural comparison of the *bs*flagellin^cent^-rTLR5^N14^ and *sd*flagellin^D1-D2^-rTLR5^N14^ (PDB ID, 3V47) complexes indicated that the lack of the hypervariable domains does not have a significant influence on the flagellin and TLR5 structures^21^. rTLR5^N14^ in complex with *bs*flagellin adopts a typical curved LRR domain structure that exhibits similar LRR curvature, radius, and twist to *sd*flagellin^D1-D2^-bound rTLR5^N14^ [root mean square deviation (RMSD), 0.50 Å]. *bs*flagellin^cent^ shares the overall shape with its corresponding region in *sd*flagellin^D1-D2^, with an RMSD value of 1.28 Å. *bs*flagellin^cent^ folds into a characteristic rod-shaped structure that is formed through extensive interactions among three long α-helices (αD1a, αD1b, and αD1c) and a coil segment containing two short β-strands (βD1a and βD1b) and loops. However, in *bs*flagellin, the coil segment and the C-terminal helix, αD1c, are directly linked through a 180° turn due to the lack of the hypervariable domains, whereas the hypervariable D2 domain is inserted between the two segments in *sd*flagellin (Figs 2a and 3a). The sharp turn of *bs*flagellin is formed by the Q188 side chain of the αD1c helix, which secures the coil segment to αD1c through a hydrogen-bond network with the main chains of D176 and T183 (Fig. 3b). The coil segment is additionally secured to the αD1a helix by hydrophobic interactions of F180 and F185 with αD1a. *bs*flagellin also exhibits structural differences at the αD1a-αD1b loop and the C-terminal part of the αD1b-βD1a loop due to one- and three-residue insertions, respectively, compared with *sd*flagellin^D1-D2^ (Fig. 3a).

**Figure 4:**
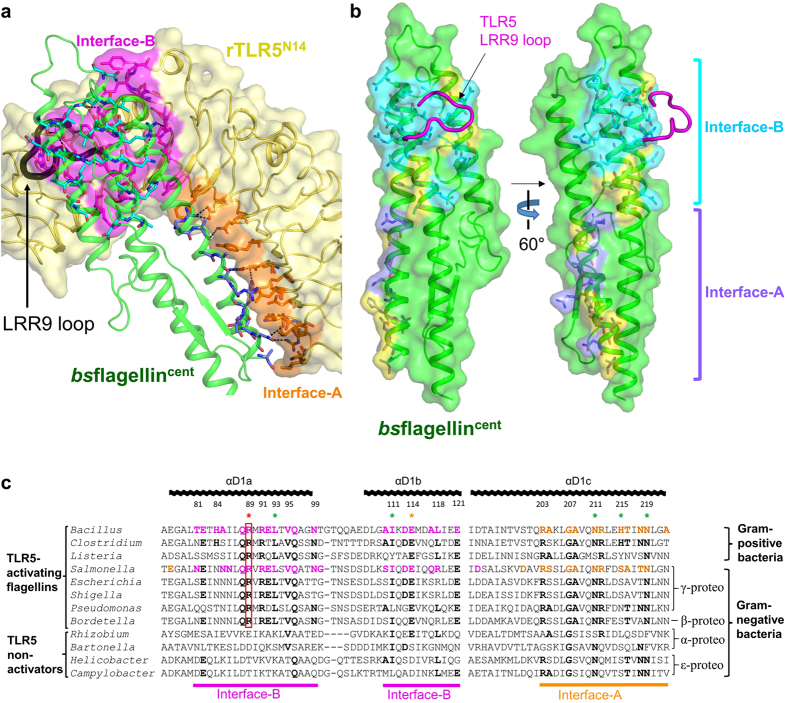
*bs*flagellin^cent^-rTLR5^N14^ interaction. (**a**) Binding interfaces of the *bs*flagellin^cent^-rTLR5^N14^ complex. rTLR5^N14^ is shown in yellow ribbons and a surface representation. Flagellin-binding TLR5 residues in interface-A and interface-B are represented by orange and magenta surfaces, respectively, and are also depicted with sticks. *bs*flagellin^cent^ is shown as green ribbons. TLR5-binding *bs*flagellin residues in interface-A and interface-B are depicted as light blue and cyan sticks, respectively. The LRR9 loop is represented by a black thick coil. (**b**) Comparison of the TLR5-binding interfaces of *bs*flagellin and *sd*flagellin. *bs*flagellin residues that are conserved with *sd*flagellin are shown as light blue (interface-A) and cyan (interface-B) surfaces with sticks. *bs*flagellin residues that are different from *sd*flagellin are colored in yellow. The TLR5 LRR9 loop is depicted as a magenta coil. (**c**) Alignment of the flagellin amino acid sequences at and around the primary binding interface. *bs*flagellin and *sd*flagellin residues in interface-A and interface-B are colored in orange and magenta, respectively, and are shown in bold. Residues of other flagellins that are identical to the *bs*flagellin residues are bolded. *bs*flagellin residues with significant effects on TLR5 activation are designated as ‘*’ color-coded as in Fig. 5c. *bs*flagellin R89 and its equivalent residues in other TLR5-activating flagellins are highlighted by a red box. The amino acid sequences are derived from *Bacillus subtilis* subspecies spizizenii strain W23, *Clostridium tyrobutyricum, Listeria innocua* serovar 6a, *Salmonella enterica* subspecies enterica serovar Dublin, *Escherichia coli* strain K-12, *Pseudomonas aeruginosa, Shigella flexneri* 2a strain 301, *Serratia marcescens* WW4, *Bordetella bronchiseptica, Helicobacter pylori* J99, *Campylobacter jejuni* subspecies jejuni, *Bartonella bacilliformis*, and *Rhizobium meliloti* strain 1021.

*bs*flagellin^cent^ forms a 1:1 complex with rTLR5^N14^ using a primary binding interface created between three long α-helices (αD1a, αD1b, and αD1c) of the *bs*flagellin D1 domain and the concave and lateral sides of TLR5 (Figs 2 and 4a). Although *bs*flagellin lacks the hypervariable domains, the *bs*flagellin^cent^-rTLR5^N14^ complex adopts a similar 1:1 architecture as the *sd*flagellin^D1-D2^-rTLR5^N14^ complex. This structural observation, combined with the similar TLR5-binding affinities of *bs*flagellin and *sd*flagellin, suggests that the D2 and D3 hypervariable domains of *sd*flagellin are dispensable for TLR5 binding.

In the *bs*flagellin^cent^-rTLR5^N14^ structure, the primary binding interface is divided into two regions, interface-A and interface-B, which are located at LRRNT-LRR6 and LRR7-LRR10 and have buried surface areas of 550 Å^2^ and 695 Å^2^, respectively (Fig. 4a). A comparative structural analysis of the *bs*flagellin^cent^-rTLR5^N14^ and *sd*flagellin^D1-D2^-rTLR5^N14^ complexes suggests that the LRR9 loop of TLR5 centered in interface-B plays a critical role in both *bs*flagellin and *sd*flagellin binding^21^. The sequences and structures of flagellin residues in interface-B that interact with the TLR5 LRR9 loop are highly conserved, whereas the residues in interface-A and in the peripheral region of interface-B show differences in sequence and structure (Fig. 4b,c).

### Identification of flagellin residues critical in TLR5 activation

**Figure 5:**
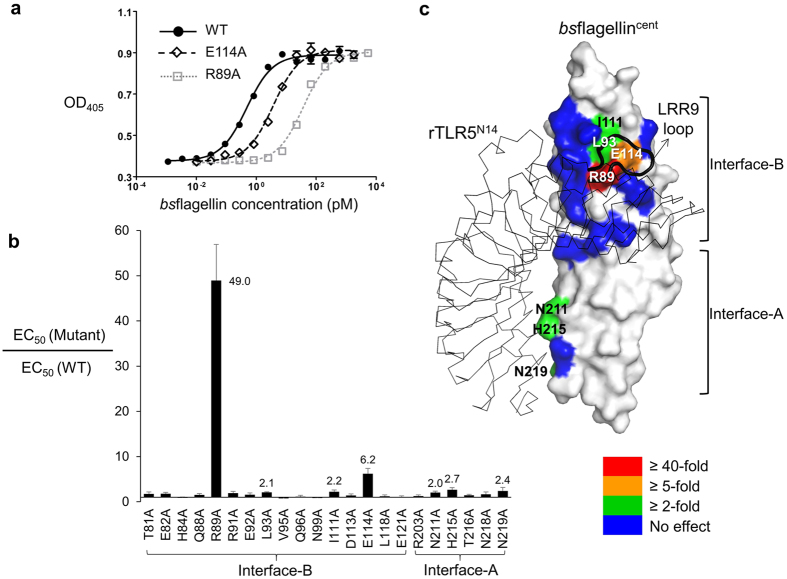
TLR5 signaling activities of *bs*flagellin alanine mutants. (**a**) TLR5 signaling activities of *bs*flagellin^WT^ and its mutants, R89A and E114A. Activities were determined using the HEK293^TLR5^ reporter cell assay. The data (means ± S.D.; n = 2) are representative of at least three independent experiments that yielded similar results. (**b**) The relative TLR5 signaling activities of *bs*flagellin alanine mutants, compared to *bs*flagellin^WT^. The data represent the means ± S.D. from at least three independent experiments. (**c**) Contributions of *bs*flagellin residues to TLR5 signaling activity that were color-coded by the effects of the mutations.

The comparison of the *bs*flagellin^cent^-rTLR5^N14^ and *sd*flagellin^D1-D2^-rTLR5^N14^ structures reveals that the flagellin D1 domain possesses a common molecular pattern for the TLR5 interaction, despite the differences in the sequences and domains between flagellins. To identify the conserved hot spot residues required for TLR5 activation, exhaustive mutational studies were performed using *bs*flagellin as a minimal TLR5-activating flagellin model that does not contain the hypervariable domains. Twenty-two non-alanine and non-glycine residues of *bs*flagellin in the TLR5-binding interface were mutated to alanine and the TLR5-stimulating activities of those alanine mutants were assessed using the HEK293^TLR5^ reporter cell assay. TLR5 activation by seven alanine mutants (R89A, E114A, L93A, I111A, N211A, H215A, and N219A) was significantly reduced, compared to the wild-type (WT) *bs*flagellin (*bs*flagellin^WT^) (Fig. 5). Interestingly, the seven residues are positionally segregated into two discrete regions of interface-A and interface-B (Fig. 5c).

**Figure 6:**
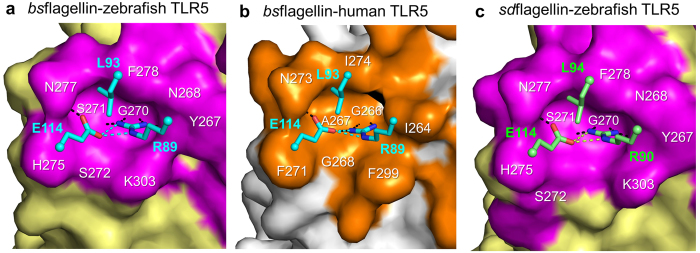
Flagellin-TLR5 interactions at and near *bs*flagellin R89A. (**a**) *bs*flagellin R89, E114, and L93 (cyan sticks) observed in the cavity formed by the zebrafish TLR5 LRR9 loop (magenta surface) in the *bs*flagellin^cent^-rTLR5^N14^ structure. Hydrogen bonds are represented by black (*bs*flagellin-to-TLR5 interactions) and cyan (*bs*flagellin-to-*bs*flagellin interactions) dashed lines. (**b**) *bs*flagellin R89, E114, and L93 (cyan sticks) observed in the cavity formed by the human TLR5 LRR9 loop (orange surface) in the *bs*flagellin-human TLR5 model. The complex model was generated by calculating a homology-based model of human TLR5 with the Modeller program^34^, combining it with the *bs*flagellin^cent^ structure obtained from the *bs*flagellin^cent^-rTLR5^N14^ structure, and applying structure idealization with the Refmac5 program^33^. Hydrogen bonds are represented by black (*bs*flagellin-to-TLR5 interactions) and cyan (*bs*flagellin-to-*bs*flagellin interactions) dashed lines. (**c**) *sd*flagellin R90, E114, and L94 (green sticks) observed in the cavity formed by the zebrafish TLR5 LRR9 loop (magenta surface) in the *sd*flagellin^D1-D2^-rTLR5^N14^ structure (PDB ID 3V47). Hydrogen bonds are represented by black (*sd*flagellin-to-TLR5 interactions) and green (*sd*flagellin-to-*sd*flagellin interactions) dashed lines.

Among the seven residues whose mutation impaired HEK293^TLR5^ activation, *bs*flagellin R89, E114, L93, and I111 cluster together in the center of interface-B (Fig. 5c). Strikingly, the *bs*flagellin^R89A^ mutant exerted drastically reduced HEK293^TLR5^ activity, with a ~49-fold lower EC_50_ value than *bs*flagellin^WT^ and was the weakest TLR5 activator among the twenty-two alanine mutants (Fig. 5). The reason for the reduced activity of the mutant can be suggested by the structural analysis. In the *bs*flagellin^cent^-rTLR5^N14^ structure, *bs*flagellin R89 protrudes and is snugly inserted into a cavity that is generated by TLR5 LRR9 loop residues (Y267, N268, G270, S271, S272, H275, N277, F278, and K303) (Fig. 6a and Supplementary Fig. S1). Furthermore, *bs*flagellin R89 extensively interacts with the wall and bottom of the TLR5 cavity. The side chain of *bs*flagellin R89 is enclosed by the side chains of TLR5 Y267, N268, S272, F278, and K303, that form the cavity wall, through van der Waals interactions and is stably hydrogen-bonded to the main-chain oxygen atoms of TLR5 cavity residues (Y267, G270, and S271). Thus, the R89A mutation in *bs*flagellin ablates the shape and chemical complementarity between *bs*flagellin R89 and the TLR5 LRR9 loop. The observed structural implications of the *bs*flagellin^R89A^ mutant were empirically tested using a competitive rTLR5^N14^-binding assay. The R89A mutation most substantially disrupted rTLR5^N14^ binding and showed ~10-fold lower activity, indicating that the impaired ability of *bs*flagellin^R89A^ to bind TLR5 directly correlates with reduced TLR5 activation (Supplementary Fig. S2).

In addition to the mutation of R89, mutations at its adjacent residues, E114 and L93, in interface-B reduced TLR5 activity by ~6-fold and ~2-fold, respectively. In the *bs*flagellin^cent^-rTLR5^N14^ structure, *bs*flagellin E114 directly and indirectly contributes to TLR5 binding. The side chain of *bs*flagellin E114 makes direct contacts with the side chains of TLR5 S272, H275, and N277 through van der Waals interactions and a hydrogen bond. In addition to the intermolecular interactions, the side chain of *bs*flagellin E114 provides intramolecular structural support for flagellin R89 through hydrogen bonds and allows it to be deeply inserted into the cavity. *bs*flagellin L93 weakly interacts with TLR5 N277 and F278 and simultaneously orients *bs*flagellin R89 and E114 toward the cavity.

To confirm that the hot spot residues, R89, E114, and L93, also play a key role in other flagellin-TLR5 systems, comparative structural and sequence analyses were performed. In a homology-based structural model of human TLR5 in complex with *bs*flagellin, the LRR9 loop of human TLR5 also forms a cavity that accommodates R89, E114, and L93 through conserved key interactions (Fig. 6b). These interactions are also recapitulated in the *sd*flagellin^D1-D2^-rTLR5^N14^ structure (Fig. 6c)^21^. Moreover, the three hot spot residues are highly conserved in TLR5-activating flagellins, but not in TLR5 non-activators (Fig. 4c). Taken together, we conclude that *bs*flagellin R89 and its neighboring residues constitute a hot spot for TLR5 activation as a conserved mechanism.

In contrast to the hot spot-forming *bs*flagellin residues in interface-B, single mutations of N211, H215, and N219 in interface-A reduced TLR5 signaling activity by only 2.0–2.7-fold and rTLR5^N14^-binding activity by 1.4–1.8-fold, compared to *bs*flagellin^WT^ (Fig. 5 and Supplementary Fig. S2). Although it is apparent that the three residues contribute to TLR5 activity in interface-A, their relative contribution to the full extent of flagellin-mediated TLR5 activation is minimal, compared to the hot spot residues of interface-B. Consistent with these observations, evolutionary restraint for amino acid sequence conservation was not observed between zebrafish TLR5 (Q80, E79, and R37) and human TLR5 (S79, G78, and C36) residues that interact with *bs*flagellin N211, H215, and N219, and thus the interactions in interface-A do not seem to be conserved.

### Contribution of the flagellin D0 domain to TLR5 binding and signaling

**Figure 7:**
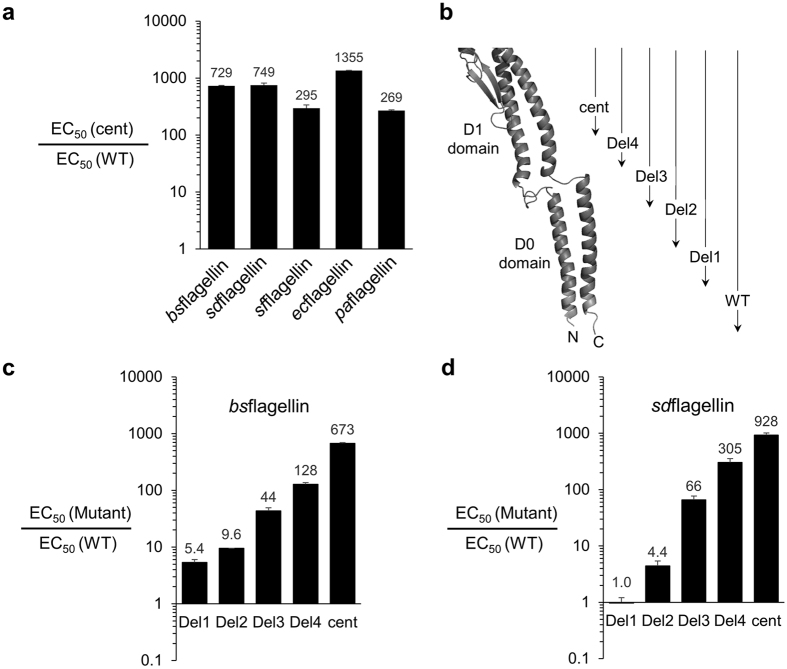
TLR5 signaling activity of *bs*flagellin and *sd*flagellin deletion mutants (means ± S.D.; n = 2). (**a**) TLR5 signaling activities of *bs*flagellin^cent^, *sd*flagellin^cent^, *pa*flagellin^cent^, *sf*flagellin^cent^, and *ec*flagellin^cent^, compared to WT flagellins. Activities were determined using the HEK293^TLR5^ reporter cell assay. (**b**) Schematic representations of the flagellin deletion constructs (Del1, Del2, Del3, Del4, and cent). The N- and C-termini of flagellin are labeled as ‘N’ and ‘C’, respectively. (**c**) TLR5 signaling activities of *bs*flagellin-Del1, Del2, Del3, Del4, and cent, compared to *bs*flagellin^WT^. Activities were determined using the HEK293^TLR5^ reporter cell assay. (**d**) TLR5 signaling activities of *sd*flagellin-Del1, Del2, Del3, Del4, and cent, compared to WT *sd*flagellin.

Our structural and mutational analyses of flagellin-TLR5 complexes highlight the hot spot residues located in the central region of the *bs*flagellin D1 domain. To examine whether the other conserved regions of flagellin are also responsible for TLR5 activation, the TLR5 signaling abilities of flagellin^cent^ proteins (*bs*flagellin^cent^, *sd*flagellin^cent^, *pa*flagellin^cent^, *sf*flagellin^cent^, and *ec*flagellin^cent^), which lack the D0 domain and the N- and C-terminal parts of the D1 domain, were determined with the HEK293^TLR5^ reporter cell assay (Figs 2a and 7a). Flagellin^cent^ proteins exhibited 269–1355-fold lower TLR5 signaling activity, compared to the full-length flagellins. To specify the exact region that affects TLR5 signaling, a series of deletion mutants were generated. The structure of the D0 domain was solely determined in the flagellar filament and consists of N- and C-terminal antiparallel helices^19^. Considering the structural integrity of the D0 domain, both N- and C-terminal residues were simultaneously removed to yield the blunt end mutants, “Del1” (*bs*flagellin residues 8–267; *sd*flagellin residues 9–496), “Del2” (*bs*flagellin residues 16–258; *sd*flagellin residues 17–487), “Del3” (*bs*flagellin residues 23–251; *sd*flagellin residues 24–480), and “Del4” (*bs*flagellin residues 33–238; *sd*flagellin residues 34–467) (Fig. 7b). The *bs*flagellin Del1, Del2, Del3, Del4, and cent mutants exhibited 5.4-, 9.6-, 44-, 128-, and 673-fold lower TLR5 signaling activity, respectively, compared to *bs*flagellin^WT^, indicating that no region is fully responsible for the enhanced TLR5 activity induced by the terminal segments of *bs*flagellin (Fig. 7c). Consistently, the Del1, Del2, Del3, Del4, and cent mutants of *sd*flagellin showed 1.0-, 4.4-, 66-, 305-, and 928-fold lower activity (Fig. 7d). Therefore, the flagellin D0 residues that contribute to TLR5 activation are scattered throughout the N- and C-terminal parts of flagellin and are not limited to a focal region.

## Discussion

Our comparative structural and mutational analyses of flagellin-mediated TLR5 activation reveal that diverse flagellins from γ-proteobacteria and Firmicutes activate TLR5 through a conserved recognition mechanism by which an arginine residue (R89 in *bs*flagellin) and its adjacent residues of the flagellin D1 domain form a complementary interaction with the LRR9 loop of TLR5. In addition to the D1 domain, the D0 domain is required to maximize flagellin-mediated cellular activity.

Not all bacterial flagellins activate TLR5. In contrast to flagellins of γ-proteobacteria and Firmicutes, flagellins from *Campylobacter jejuni* and *Helicobacter pylori* that belong to ɛ-proteobacteria do not induce TLR5 signaling and thus can escape from TLR5-mediated immune surveillance^26^. The major functional difference between TLR5-activating flagellins and non-activators can be ascribed to sequence variations. In *C. jejuni* and *H. pylori* flagellins, hot spot residues (*bs*flagellin R89 and E114) are replaced with threonine and aspartate residues, respectively, which would fail to make complementary contacts with the TLR5 LRR9 loop. In addition, *C. jejuni* and *H. pylori* flagellins share only 21–26% sequence identity with *bs*flagellin in binding interface-B, which may be one reason for the lack of the TLR5 activation by *C. jejuni* and *H. pylori* flagellins. *C. jejuni* flagellin may be evolutionarily endowed with the divergent sequence potentially due to the distinct organization of the flagellar filament. The *Salmonella* filament consists of eleven protofilaments. *Salmonella* flagellin residues located between protofilaments are highly conserved and are also involved in the TLR5 interaction^19^. However, the flagellar filament of *C. jejuni* is composed of seven protofilaments and, thus, evolutionary restraint on the TLR5-binding residues might have been substantially reduced in *C. jejuni* flagellin^27^.

The deletion of the N- and C-terminal regions in flagellins reduced TLR5 stimulation in the reporter cell assay (Fig. 7a). Because the terminal regions of *sd*flagellin were previously reported to be dispensable for TLR5 binding, these regions may affect TLR5 activation by promoting TLR5 dimerization or interaction with unknown co-receptors^21^. Interestingly, *ec*flagellin was previously shown to interact with alginate, an anionic polysaccharide. Thus, the terminal regions of flagellin may be involved in recruiting flagellin to negatively charged sugars, such as sialic acid, appended to TLR5, or to phospholipids on the cell membrane where TLR5 is located^28^. However, structural studies to visualize the interaction would be technically difficult due to the diverse conformations of the terminal regions. Although the D0 domain of flagellin is ordered and folded into two α-helices in the flagellar filament, the terminal regions that include the D0 domain are disordered in solution^19^,^29^,^30^. Moreover, the terminal part of the D1 domain changes its conformation depending on the environment. For example, the C-terminal part of the D1 domain adopted an extended structure instead of the α-helix observed in the flagellar filament^21^.

*bs*flagellin contains only D0 and D1 domains and is one of the shortest flagellins. Although *bs*flagellin lacks the hypervariable domains, it exhibits similar TLR5-binding affinity and TLR5 signaling activity to other flagellins. Thus, *bs*flagellin provides an alternative avenue for flagellin-based anti-radiation therapeutics and anti-pathogenic vaccines that do not induce unwanted adaptive immunity or other unexpected cellular toxicity. CBLB502 was developed as an anti-radiation therapeutic by removing the hypervariable D2 and D3 domains from *sd*flagellin^14^. However, CBLB502 seems to be relatively unstable, compared to the full-length *sd*flagellin protein, given that CBLB502 forms inclusion bodies upon expression in an *E. coli* expression system and the CBLB502 protein tends to degrade during purification and storage. We believe that the instability of CBLB502 results from the addition of the partial sequence of the D2 domain and 16 artificial residues into the middle of the D1 domain between the coil segment and αD1c, without consideration of the structural integrity and protein folding properties. The stability of the CBLB502 protein can be improved by mimicking the loop structure of *bs*flagellin that directly links the coil segment and αD1c. Alternatively, *bs*flagellin as a natural flagellin or *bs*flagellin D1 fused to *sd*flagellin D0 can be developed as therapeutics.

## Methods

### Expression and purification of the flagellin and rTLR5^N14^ proteins

DNAs that encode full-length flagellin and its partial fragments were generated by PCR from bacterial genomic DNAs. PCR products were digested with a set of two restriction enzymes, *Bam*HI/*Sal*I, *Bgl*II/*Sal*I, or *Bam*HI/*Xho*I, and ligated into a *Bam*HI/*Sal*I-digested pET49b vector that had been designed to express proteins with an N-terminal His_6_ tag and a thrombin cleavage site. Alanine mutations were performed using the QuikChange site-directed mutagenesis protocol (Agilent). Clones were confirmed by DNA sequencing.

The recombinant flagellin protein was over-expressed in *E. coli* BL21 (DE3) cells. Protein expression was induced at an OD_600_ of ~0.8 using 1 mM IPTG at 37 °C for 3 hours. Cells were collected by centrifugation and lysed by sonication. The lysate was cleared by centrifugation and the supernatant was applied to a Ni-NTA affinity chromatography column (Qiagen) for initial purification. The flagellin protein was eluted using 250 mM imidazole and dialyzed against 20 mM Hepes, pH 7.4 or 20 mM Tris, pH 8.0. The N-terminal tag was cleaved from flagellin by incubating the protein with thrombin for 3 hours at 18 °C. The resulting flagellin protein was further purified by anion exchange chromatography using a Mono Q column (GE Healthcare).

A fusion protein, rTLR5^N14^, between the N-terminal fragment of zebrafish TLR5 (LRRNT to LRR14) and the C-terminal fragment of hagfish variable lymphocyte receptor (VLR) was expressed and purified as previously described^21^,^25^. rTLR5^N14^ is appended to the C-terminal thrombin cleavage site, Strep-tag II (WSHPQFEK), and His_6_ tag to facilitate purification. rTLR5^N14^ was expressed in the baculovirus expression system and initially purified using Ni-NTA and Strep-Tactin columns. The C-terminal tags of rTLR5^N14^ were removed with thrombin and further purified by gel-filtration chromatography to yield a tag-free rTLR5^N14^.

For the competitive binding assay, rTLR5^N14^ appended to the C-terminal Avitag (rTLR5^N14^-Avitag) was expressed and purified in the baculovirus expression system similarly to the rTLR5^N14^ protein. The rTLR5^N14^-Avitag was biotinylated using BirA biotin ligase (Avidity).

### Determination of the TLR5 signaling activity of flagellin

The TLR5 signaling potency of flagellin was measured by monitoring NF-κB activity in HEK293^TLR5^ cells (InvivoGen) that express the SEAP reporter gene under the control of TLR5-stimulated NF-κB. As described in manufacturer’s protocol, the HEK293^TLR5^ cells were grown in DMEM that contains 4.5 mg/ml glucose, 2 mM glutamine, 10% fetal bovine serum, 15 μg/ml blasticidin, and 100 μg/ml Zeocin. For the flagellin-mediated TLR5 activation assay, the flagellin protein was serially diluted in DMEM and incubated with 25,000 HEK293^TLR5^ cells in 200 μl for 12 hours at 37 °C. To measure SEAP activity, 50 μl of the culture was incubated with 20 μl of p-nitrophenyl phosphate (an alkaline phosphatase substrate, Sigma-Aldrich) and 80 μl of 20 mM Hepes, pH 7.4 and 150 mM NaCl for 30 minutes at 37 °C. SEAP activity was determined by spectrophotometry at 405 nm.

### Analysis of the interaction between flagellin and rTLR5^N14^

The direct interaction between flagellin and rTLR5^N14^ was monitored by native PAGE and gel-filtration chromatography analyses. Purified flagellin and rTLR5^N14^ proteins were incubated at 18 °C for 30 minutes and analyzed for complex formation. Native PAGE was performed using 6% polyacrylamide gels at room temperature for 1.5 hours at 100 V. Gel-filtration chromatography was performed using a Superdex 200 10/300 column (GE Healthcare).

The relative binding affinities of the flagellins and their mutants for TLR5 were determined using a competitive binding assay. First, 96-well plates were coated with 50 μl of 0.2 μg/ml *bs*flagellin in PBS for 24 hours. Each well was washed three times using PBS containing 0.05% Tween 20 (PBS-T) and blocked with 1% bovine serum albumin in PBS-T (blocking solution). Next, 50 μl of the serially diluted flagellin protein and 50 μl of 0.02 μg/ml biotinylated rTLR5^N14^-Avitag in the blocking solution were incubated in each well for 48 hours at 4 °C. Then, 100 μl of horseradish peroxidase-conjugated streptavidin (Thermo Fisher) in the blocking solution was added and incubated for 1 hour at room temperature. Finally, 100 μl of tetramethylbenzidine (Thermo Fisher) was added to each well. After a 3-minute incubation, the reaction was stopped by the addition of 100 μl of 1 M sulfuric acid. The light absorbance was measured at 450 nm.

### Crystallization and X-ray diffraction data collection

To crystallize the flagellin-TLR5 complex, rTLR5^N14^ and *bs*flagellin^cent^ were employed. rTLR5^N14^ was incubated with *bs*flagellin^cent^ for 1 hour at 18 °C. The resulting complex was purified by gel-filtration chromatography using a Superdex 200 16/600 column (GE Healthcare) and concentrated for crystallization.

The *bs*flagellin^cent^-rTLR5^N14^ complex was crystallized using the sitting-drop vapor-diffusion method in 1 μl drops that contained 4.65 mg/ml protein complex, 17.5% (*w/v*) PEG 300, and 50 mM sodium acetate, pH 4.4 at 18 °C. Crystals were flash-frozen at 100 K and X-ray diffraction was performed at beamlines 5C and 7A of the Pohang Accelerator Laboratory (Korea). Diffraction data were processed using HKL2000^31^. Data collection statistics are shown in Supplementary Table S1.

### Structure determination

The crystal structure of the *bs*flagellin^cent^-rTLR5^N14^ complex was determined by molecular replacement using the search models, rTLR5^N14^ and the *Salmonella* flagellin D1 domain (PDB ID, 3V47). Iterative model building and refinement were performed using the Coot^32^ and Refmac5^33^ programs, respectively, to obtain the final structural model of the *bs*flagellin^cent^-rTLR5^N14^ complex. Refinement statistics are shown in Supplementary Table S1.

### Data Availability

The atomic coordinates and structure factors for the bsflagellincent-rTLR5N14 complex (PDB ID 5GY2) have been deposited in the Protein Data Bank, www.pdb.org.

## Additional Information

**How to cite this article**: Song, W. S. *et al*. A conserved TLR5 binding and activation hot spot on flagellin. *Sci. Rep.* **7**, 40878; doi: 10.1038/srep40878 (2017).

**Publisher's note:** Springer Nature remains neutral with regard to jurisdictional claims in published maps and institutional affiliations.

## Supplementary Material

Supplementary Information

## Figures and Tables

**Table 1:** TLR5 signaling activities and rTLR5^N14^-binding abilities of flagellins.

Flagellin	EC_50_ (pM) for TLR5 signaling	IC_50_ (pM) for rTLR5^N14^ binding
*bs*flagellin	1.18 ± 0.34	204 ± 15
*sd*flagellin	0.57 ± 0.11	289 ± 73
*sf*flagellin	1.57 ± 0.55	414 ± 34
*ec*flagellin	1.13 ± 0.14	517 ± 7
*pa*flagellin	1.68 ± 0.42	673 ± 163

EC_50_ values for the TLR5 signaling activity were determined using the HEK293^TLR5^ cell assay and IC_50_ values for the rTLR5^N14^-binding abilities were calculated from the competitive rTLR5^N14^-binding assay. The data are shown as the means ± S.D.; n ≥ 3.
