# Supplementary material for: A conserved TLR5 binding and activation hot spot on flagellin
Source: Sci Rep. 2017 Jan 20;7:40878. doi: 10.1038/srep40878 (PMC5247705; doi:10.1038/srep40878)
Supplement: Supplementary Information [file srep40878-s1.pdf]

A conserved TLR5 binding and activation hot spot on flagellin

- Supplementary Figures S1 and S2
- Supplementary Table S1

Wan Seok Song[1], Ye Ji Jeon[1], Byeol Namgung[1], Minsun Hong[2 *], Sung-il Yoon[1,3 *]

[1]Division of Biomedical Convergence, College of Biomedical Science, Kangwon National University, Chuncheon 24341, Republic of Korea

[2]Division of Biological Science and Technology, Yonsei University, Wonju 26493, Republic of Korea.

[3]Institute of Bioscience and Biotechnology, Kangwon National University, Chuncheon 24341, Republic of Korea

* To whom correspondence should be addressed.

- Sung-il Yoon (E-mail: sungil@kangwon.ac.kr, Phone: +82-33-250-8385, Fax: +82-33-259-5643, Address: 1 Kangwondaehak-gil, Biomedical Science building A-204, Chuncheon 24341, Republic of Korea)
- Minsun Hong (E-mail: minsunhong@yonsei.ac.kr, Phone: +82-33-760-2243, Fax: +82-33-760-2183, Address: 1 Yonseidae-gil, Mirae building #303, Wonju 26493, Republic of Korea)

Supplementary Table S1. Crystallographic statistics of the *bs*flagellin^{cent}-rTLR5^{N14} structure.

	*bs*flagellin^{cent}-rTLR5^{N14}
**Data collection**	
Space group	$P2_1$
Cell parameters	a = 78.15 Å, b = 54.28 Å, c = 165.98 Å
	α = 90.00°, β= 98.62°, γ= 90.00°
Wavelength (Å)	0.9796
Resolution (Å)	30.00 - 2.10
Highest resolution (Å)	2.18 - 2.10
No. observations	274,773
No. unique reflections	80,115
R_{merge} (%)[a]	9.1 (51.4)[b]
I/sigma	16.8 (2.7)[b]
Completeness (%)	98.1 (95.2)[b]
Redundancy	3.4 (3.3)[b]
**Refinement**	
Resolution (Å)	30.00 - 2.10
No. of reflections (working)	75,311
No. of reflections (test)	3,987
R_{cryst} (%)[c]	19.5
R_{free} (%)[d]	23.6
Average B-value (Å^2)	33.3
No. protein atoms	9,429
No. water molecules	465
No. ligand atoms	140 (N-linked glycan)
	26 (Polyethylene glycol)
RMSD bonds (Å)	0.016
RMSD angles (°)	1.51
Ramachandran[e] (favored)	96.1%
(outliers)	0%

[a]$R_{merge} = \Sigma_{hkl}\Sigma_i \mid I_i(hkl) - \langle I(hkl)\rangle \mid / \Sigma_{hkl}\Sigma_i I_i(hkl)$

[b]Numbers in parenthesis were calculated from data of the highest resolution shell.

[c]$R_{cryst} = \Sigma\| |F_{obs}|-|F_{calc}| \| / \Sigma|F_{obs}|$ where F_{calc} and F_{obs} are the calculated and observed structure factor amplitudes, respectively

[d]R_{free} = as for R_{cryst}, but for 5 % of the total reflections chosen at random and omitted from refinement

[e]Calculated using MolProbity (http://molprobity.biochem.duke.edu).

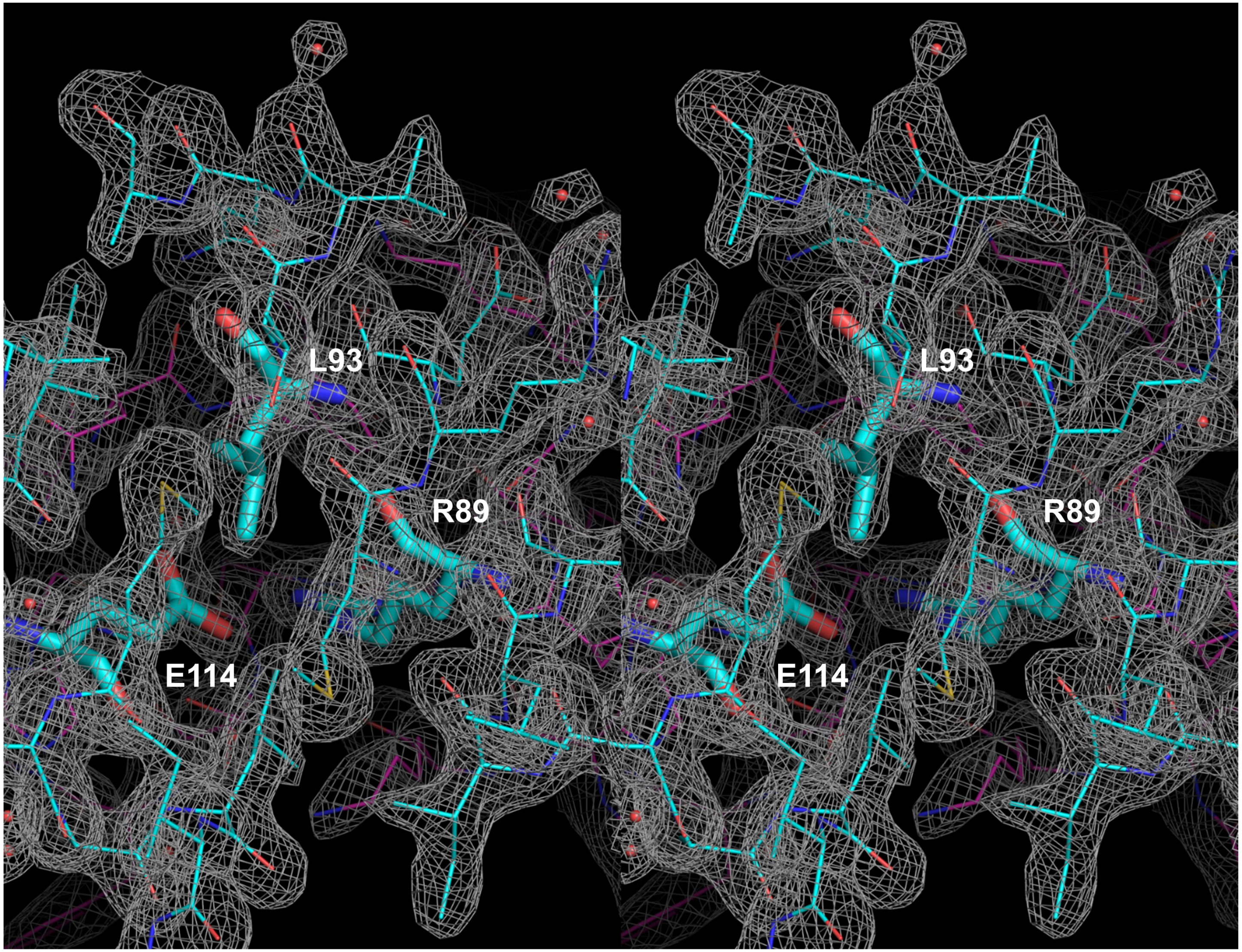

Supplementary Figure S1. Stereo view showing the intermolecular interactions of *bs*flagellin R89, E114, and L93 with TLR5 residues in the *bs*flagellin^{cent}-rTLR5^{N14} structure. *bs*flagellin^{cent} and rTLR5^{N14} are depicted as cyan and magenta lines, respectively. *bs*flagellin R89, E114, and L93 are represented by cyan sticks. Water molecules are shown as red spheres. The gray mesh represents the 2Fo-Fc map contoured at 1.3σ.

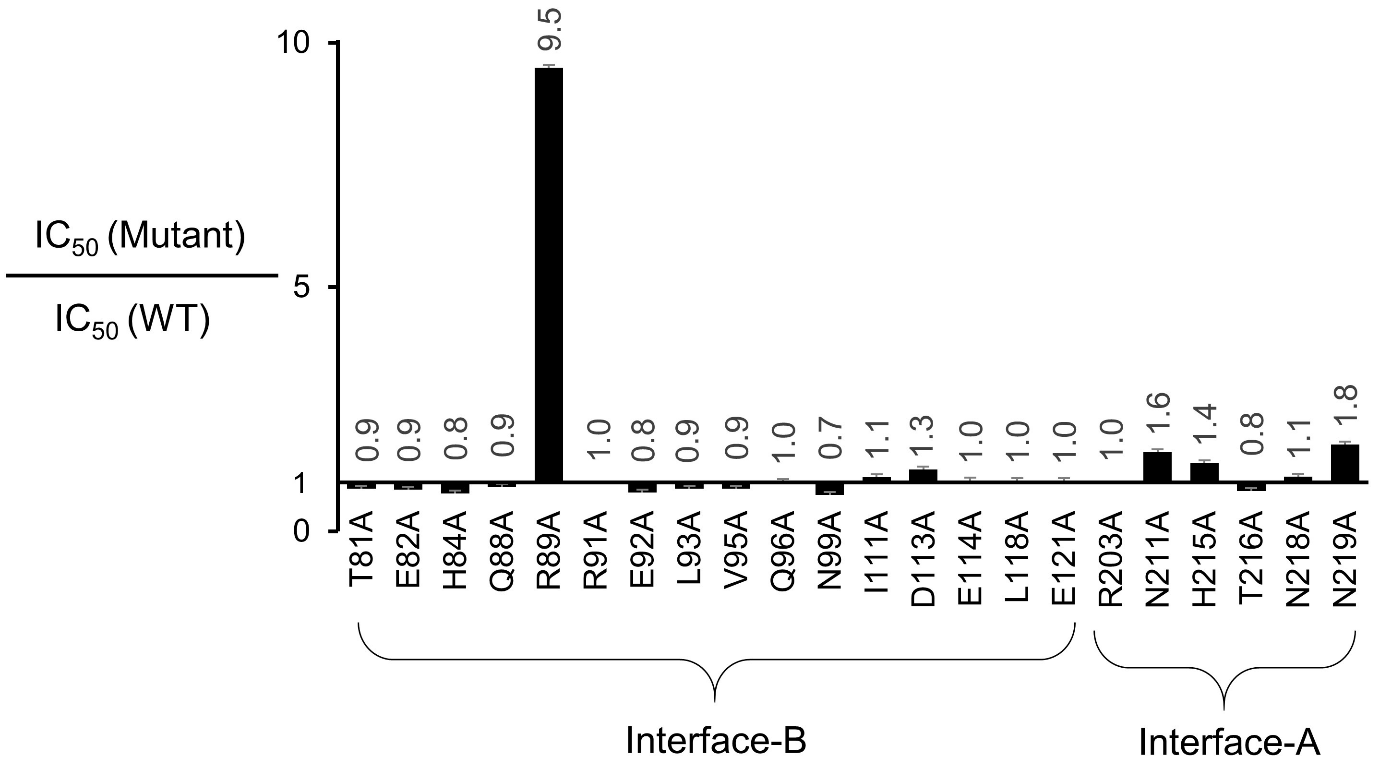

Supplementary Figure S2. Relative TLR5-binding activity of the *bs*flagellin alanine mutants, compared to *bs*flagellin^{WT}. Activity was determined using the competitive rTLR5^{N14}-binding assay (means ± S.D.; n ≥ 2). Although the rTLR5^{N14}-binding assay was less sensitive to the mutations than the HEK293^{TLR5} reporter cell assay, the binding assay obviously showed that *bs*flagellin R89 functions as a key determinant for TLR5 binding.
